# Motivations to Enter and Remain in Community Nursing: Implications for Workforce Sustainability

**DOI:** 10.1155/jonm/7221720

**Published:** 2026-09-22

**Authors:** Cornelia Feichtinger, Helmut Beichler, Elisabeth Rappold, Igor Grabovac

**Affiliations:** ^1^ Center for Applied Nursing Research, Department of Applied Nursing Sciences, University of Applied Sciences Campus Vienna, Vienna, Austria; ^2^ Vienna Healthcare Group, School of Nursing, University of Applied Sciences Campus Vienna, Vienna, Austria; ^3^ Department for Health Professions and Long-Term Care, Austrian National Public Health Institute (GÖG), Vienna, Austria; ^4^ Department of Social and Preventive Medicine, Centre for Public Health, Medical University of Vienna, Vienna, Austria, meduniwien.ac.at

**Keywords:** community nursing, motivation, retention

## Abstract

**Aim:**

To explore what motivates nurses to enter and remain in community nursing within Austria’s national pilot project and to examine how autonomy, competence and relatedness shape their professional experiences and retention intentions.

**Background:**

Community nursing has been introduced in Austria as an innovative, preventive, community‐based model of care. As nursing systems face growing workforce shortages, understanding why nurses choose to transition into and remain in new nursing roles is critical for workforce sustainability and effective service implementation.

**Methods:**

A qualitative study guided by self‐determination theory was conducted. Semistructured interviews were carried out with 15 community nurses working in Austria’s community nursing pilot project. Data were analysed using qualitative content analysis, combining inductive coding with a theory‐informed analytical framework.

**Results:**

Nurses reported high levels of motivation when their needs for autonomy, competence and relatedness were met. Autonomy was supported through flexible scheduling, independent decision‐making and opportunities to shape emerging service structures. Competence was strengthened through direct client contact, diverse responsibilities, interprofessional collaboration and professional development. Relatedness emerged from strong connections with local communities, professional networks and interdisciplinary partners. While intrinsic motivation was driven by meaningful work and contribution to preventive care, extrinsic motivation was strongly influenced by dissatisfaction with previous working conditions.

**Conclusions:**

Community nursing offers an attractive alternative career pathway for nurses by providing working conditions that support psychological needs and professional fulfilment. However, the discontinuation of the pilot project threatens the long‐term sustainability of this role.

**Implications for Nursing Management:**

Nursing managers and policymakers should prioritize work environments that support autonomy, competence and relatedness to enhance nurse retention. Integrating community nursing into long‐term workforce and primary care strategies may strengthen workforce stability and support sustainable, community‐based care delivery.

## 1. Introduction

The Austrian healthcare system has long been characterized by a curative and physician‐led orientation, with services predominantly delivered through hospitals and specialized medical practices [1]. Preventive and community‐based interventions historically received limited emphasis, resulting in a system that is highly effective at managing acute conditions but less equipped to promote long‐term population health. This hospital‐centred approach contributes to Austria’s comparatively high health expenditures: In 2022, total health spending reached €49.9 billion, equivalent to 11.2% of the gross domestic product [2]. Despite this substantial investment, the number of healthy life years at birth was only 60.5 years for women and 60.3 years for men, which is below the EU average of approximately 63.3 years for women and 62.8 years for men [3]. This discrepancy points to a mismatch between spending and outcomes, highlighting the need for stronger preventive measures and community‐oriented care [4].

International comparisons further underscore these systemic gaps. In many Scandinavian and northern European countries, robust primary healthcare systems and community‐based models are the backbone of population health strategies. These systems offer coordinated, multidisciplinary care closer to where people live, reducing avoidable hospitalizations and promoting healthier ageing. Austria’s primary care sector, by contrast, remains fragmented, with slow progress in implementing multidisciplinary primary care centres [5]. As a result, many patients continue to rely on hospital‐based acute services, leading to inefficiencies and avoidable use of high‐cost resources. This structural imbalance becomes increasingly problematic in light of demographic changes: The proportion of adults aged 65 years and over is projected to rise steadily, reaching 27.2% of the total population by 2050 [6]. Many of these older adults express a preference to remain in their homes and communities rather than transition into institutional care, a trend that is consistent with international findings on ageing in place [7–9]. Together, these factors call for innovative models that strengthen primary care, shift resources toward prevention, and support people in living independently for as long as possible.

## 2. Background

To address these challenges, Austria launched an EU‐funded community nursing pilot project in 2022 [10]. The program targeted community‐dwelling individuals aged 75 years and older, as this group was identified as having the highest risk of care dependency and unmet preventive needs and aimed to improve access to preventive and health‐promoting services, coordinate care and offer support for navigating the healthcare system. Individuals are referred to community nursing through several pathways including self‐referral, referral by general practitioners and referral by other community stakeholders, with the specific pathway used varying by region [11]. In addition to the primary target group, community nursing services also addressed informal caregivers and other population groups identified through regional needs assessments, allowing flexible adaptation to local demands; these may include individuals with chronic conditions, people at risk of frailty or social isolation and vulnerable populations such as socially disadvantaged individuals, as well as the broader community [11]. As an additional service, it was designed to complement existing mobile and home care services rather than replace them. Prior to the introduction of the community nursing pilot project, Austria did not have a standardized, nationwide model of community‐based nursing comparable to those established in countries such as the Netherlands, Denmark and Norway. While mobile care and home nursing services existed, these were primarily task‐oriented and focused on direct care provision rather than prevention, health promotion and case coordination Austrian National Public Health Institute [12].

Within this context, community nurses, who are registered nurses, act as crucial links between clients, physicians, allied health professionals, social services and municipal authorities. Their work encompasses health education, early identification of risk factors, chronic disease management and guidance for informal caregivers [11]. By promoting health literacy and empowering older adults, community nurses may help reduce hospital admissions and improve quality of life [13–15]. These objectives are closely aligned with the World Health Organization’s call to strengthen primary healthcare and community‐based nursing as part of the global strategy towards universal health coverage [16].

However, the success of this initiative depends not only on its design but also on the professionals who deliver it. Austria, like many countries, faces persistent nursing workforce challenges. Although it has approximately 11 nurses and midwives per 1000 inhabitants, which is above the EU average, this density remains modest compared to neighbouring countries such as Germany (almost 12 per 1000) and Switzerland (over 18 per 1000) [17]. By comparison, Austria has an above‐EU‐average physician density of approximately 5.5 doctors per 1000 inhabitants [5, 18]. Within the community setting, community nurses and general practitioners are intended to work in a complementary, collaborative relationship: community nurses focus on prevention, health promotion and care coordination, while general practitioners retain responsibility for medical diagnosis and treatment, with referral pathways between the two professions forming a key element of the model. At the same time, the workforce is ageing, and nurse attrition is a growing concern. High workloads, limited career progression, emotional exhaustion and moral distress have been identified as significant contributors to nurse turnover [19, 20]. International studies suggest that improvements in the psychosocial work environment could reduce turnover substantially, for example, by up to 44% in Danish hospital settings shown by Kovacevic et al. [21]. Without targeted strategies to attract and retain nurses, Austria risks facing critical staff shortages that may jeopardize service provision.

A substantial body of qualitative research has investigated the factors that drive nurses out of the profession and those that support retention. A qualitative meta‐aggregation by Bahlman‐van Ooijen et al. [22], synthesizing nine qualitative studies from multiple countries, identified emotional exhaustion, moral distress, inadequate staffing, lack of organizational support and limited career development as the central reasons nurses leave. Crucially, the same review found that a sense of meaningful work, collegial relationships and professional recognition were among the few factors capable of sustaining commitment to the profession over time. These findings align with a qualitative study focussing on German nurses by Roth et al. [23], who found that nurses’ decisions to leave nursing were driven by limited career prospects, workplace pressures and a poor public image of nursing, while professional pride, recognition and opportunities for professionalization acted as pull factors encouraging them to stay. Together, these studies demonstrate that the decision to leave or remain in nursing is rarely the result of a single factor; rather, it reflects a complex interplay of push and pull forces operating across individual, organizational and systemic levels [24].

What nurses are motivated toward, rather than simply away from, has received comparatively less qualitative attention. Hörberg et al. [25] explored the simultaneous relationship between workplace motivation and intention to leave among experienced Swedish nurses, finding that motivation was sustained primarily by meaningful patient relationships, professional development opportunities and a sense of competence, while its absence was the strongest predictor of turnover intention. Similarly, Messineo et al. [26] applied self‐determination theory (SDT) to the motivations of nursing students, demonstrating that autonomous motivation, which is driven by genuine interest and personal values, was associated with stronger commitment to the profession than controlled or externally driven motivations. Falomo et al. [27] further showed that nurses who stayed in their role despite considering leaving did so largely through intrinsic factors: a sense of professional calling, attachment to their team and the perceived irreplaceability of their contribution to patient care.

The majority of this research has focused on hospital and institutional care settings, where working conditions, hierarchies and task structures differ from community‐based roles. Qualitative evidence specifically examining what motivates nurses to choose a community nursing role, particularly in contexts where this role is newly established, time‐limited and structurally uncertain, remains sparse. This gap is particularly relevant in the Austrian context, where community nursing was introduced as a pilot project with no established professional tradition, requiring nurses to actively commit to a role whose long‐term future was uncertain from the outset.

Community nursing has the potential to offer a compelling alternative career pathway for nurses. The model emphasizes autonomy, flexibility and person‐centred care, characteristics that have been associated with higher job satisfaction and stronger retention [28, 29]. Beyond supporting workforce sustainability, it also contributes to public health goals by focussing on prevention, early intervention and empowerment of older adults, thus potentially increasing healthy life years and reducing healthcare costs in the long term [30, 31]. Despite these benefits, there remains a lack of empirical research examining why nurses chose to leave secure positions in hospitals or long‐term care facilities to take on the pioneering role of community nurse in a time‐limited pilot project. Understanding their motivations is essential for workforce planning, recruitment and the design of supportive working environments.

This study is guided by SDT, a widely applied framework for understanding human motivation [32–34]. SDT posits that three basic psychological needs must be satisfied to foster intrinsic motivation: autonomy, competence and relatedness. When these needs are met, individuals are more likely to engage in activities with intrinsic motivation, out of interest, enjoyment or personal value, rather than relying solely on external rewards or pressures. SDT also distinguishes between intrinsic and extrinsic motivation. While intrinsic motivation tends to produce more sustainable engagement and satisfaction, extrinsic motivation can initiate behaviour change when supported by internalization processes, such as aligning external expectations with personal values [33].

SDT has been applied across multiple contexts in health, education and organizational psychology to explain well‐being, persistence and performance. By framing this study within SDT, it becomes possible to identify which aspects of community nursing fulfil or frustrate these needs and how this influences nurses’ decisions to enter this emerging field. These insights can inform workforce development strategies and support the long‐term sustainability of community‐based nursing services in Austria.

## 3. The Study

### 3.1. Aim(s)/Objective(s)/Research Question

The aim of this study is to explore what motivates nurses to enter and remain in community nursing within Austria’s national pilot project and to examine how autonomy, competence and relatedness shape their professional experiences and retention intentions.

## 4. Methods/Methodology

### 4.1. Design

A qualitative descriptive design was employed, which allowed the research team to explore, in participants’ own terms, what motivates nurses to enter and remain in community nursing [35]. Data were collected and analysed using qualitative content analysis [36, 37], which served as the data collection and analysis method within this descriptive design, chosen to gain in‐depth insight into participants’ roles, challenges and experiences within the community.

### 4.2. Study Setting and Recruitment

Twenty eight community nurses employed within the community nursing pilot project across six regions of Austria were invited to participate between March 2023 and June 2023. Of these, 15 agreed to take part, while 13 declined, without providing a specific reason. The data collection process was carried out through guided interviews [38]. Most interviews (*n* = 11) were conducted in person, while four were conducted online via Zoom to accommodate geographical distance, illness‐related absences and scheduling flexibility.

The 15 participating community nurses were aged between 25 and 55 years (*M* = 38.2 and SD = 7.49) and were predominantly female (*n* = 14), with one male participant. Participants had a mean of 13.5 years (SD = 6.7 and range 1.9–24.7) of professional nursing experience at the time of data collection (reference date 30 June 2023). Three participants held a Bachelor’s degree (e.g., in nursing science or in health and nursing management), and one held a Master’s degree; eight participants had completed additional specialized postbasic training (e.g., wound management, mental health nursing, and advanced nurse practice); the remaining three participants had no additional postbasic qualification.

### 4.3. Data Collection

Data were collected through individual, semistructured interviews rather than focus groups. This approach was chosen because the study aimed to capture each nurse’s individual professional trajectory and experience, and because participants’ career backgrounds varied considerably; an individual format allowed these distinct narratives to be explored in depth rather than potentially flattened within a group setting. Individual interviews were also conducted in conjunction with a one‐day shadowing period per nurse, making one‐on‐one scheduling more practical, and were logistically more feasible given that participants were dispersed across different regions of Austria, eliminating the need for travel and allowing interviews to be scheduled within nurses’ existing working days. Interviews were conducted during nurses’ paid working hours and scheduled in advance. All interviews were audio‐recorded and subsequently transcribed verbatim. The interview guide was developed with partial input from field experts to ensure that the questions were practical and closely aligned with the research topic. It was semistructured [39], beginning with an open question on participants’ motivation to apply (“Why did you apply as a Community Nurse? How did you imagine the profession?”), followed by questions on daily tasks and routines (e.g., “Could you tell me about a typical case from your work?”), professional barriers and the support needed to address them, network partners and interprofessional collaboration, and participants’ outlook for the future of community nursing in Austria. Interview duration ranged from 17 to 63 min, with most interviews exceeding 30 min; the shortest interview reflected a participant with a comparatively narrow range of responsibilities at the time of data collection, rather than a methodological limitation. Data collection continued until no substantively new information emerged from subsequent interviews, with core themes recurring across participants, indicating that thematic saturation had been reached at *n* = 15.

### 4.4. Data Analysis

The collected data were analysed using qualitative content analysis following the approach of Kuckartz and Rädiker [37], combining deductive and inductive elements: deductive categories derived from SDT were complemented by categories developed inductively from within participant’ accounts. The analysis proceeded in three steps. First, coding was entirely inductive: Categories were developed directly from the interview transcripts without reference to SDT, so that content not anticipated by the theoretical perspective could also be captured. Second, once this inductive category system was finalized, the resulting categories were mapped onto the three psychological needs proposed by SDT (autonomy, competence and relatedness) as a separate, subsequent analytical step. Third, connections were established between these needs, the categories and intrinsic and extrinsic forms of motivation. An illustrative excerpt of this coding process shows the progression from raw meaning units to codes, categories, and SDT dimensions. MAXQDA software (Version 24.3.0) was used throughout to organize transcripts, manage the coding system and document coding decisions, supporting consistency and traceability; it did not itself determine the analytic categories, which were developed and interpreted by the research team.

### 4.5. Ethical Considerations

Prior to the interviews, all participants received written participant information outlining the study aims, procedures, voluntary nature of participation and their right to withdraw at any time without giving reason and without any disadvantage to their employment; this information was also explained orally, after which written informed consent was obtained. Additionally, the research project underwent review and received approval from the ethics committee of the University of Applied Sciences FH Campus Wien (EK 113/2023), in accordance with the Declaration of Helsinki and the ICN Code of Ethics for Nurses. Interviews were audio‐recorded and transcribed verbatim. Transcripts were pseudonymized using a letter‐based coding system; place names, organizations and other potentially identifying details were anonymized following a standardized anonymization protocol developed for this study. This study was cross‐sectional, not longitudinal; participants were interviewed once, and audio recordings were retained after transcription (rather than deleted) to allow verification of transcript accuracy and reanalysis if required, in line with institutional data retention requirements and good scientific practice. The key linking codes to participant identities are stored securely and are accessible only to the study lead. All data, including audio recordings, are stored on password‐protected devices and will be archived for 10 years following the completion of the project.

### 4.6. Rigour and Reflexivity

This study was conducted by researchers with professional backgrounds in nursing and health services research. The research team’s prior experience with community‐based care and nursing workforce issues informed the study design and facilitated a contextualized understanding of participants’ accounts. At the same time, the team remained attentive to the potential influence of these perspectives on data collection and interpretation.

Reflexivity was supported through ongoing critical reflection throughout the research process, including discussions within the research team regarding emerging interpretations, assumptions and alternative explanations. Where appropriate, emerging findings were discussed within the research team to enhance analytic depth and to challenge individual interpretations. Analytic decisions were documented, and interpretations were continuously checked against the original interview material to ensure that findings were grounded in participants’ accounts rather than researchers’ preconceptions.

Rigour was further enhanced through the systematic application of qualitative content analysis, transparent documentation of analytic steps and the use of qualitative data analysis software (MAXQDA) to support consistency and traceability of coding. These strategies contributed to the credibility and trustworthiness of the findings, following established criteria for trustworthiness in qualitative research [40].

## 5. Findings

**FIGURE 1:**
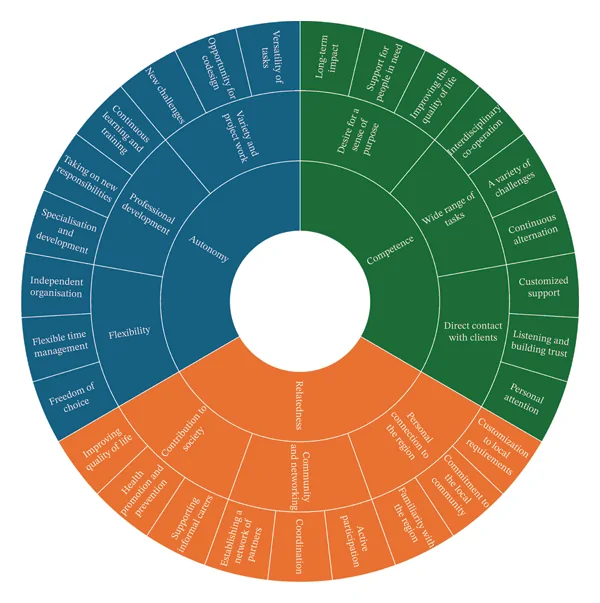
Motivational drivers in community nursing: linking inductive categories to psychological needs and motivation types. Based on a two‐step analysis, inductive coding and assignment to self‐determination theory needs (autonomy, competence and relatedness).

This sunburst graph (Figure 1) illustrates the three fundamental psychological needs defined by SDT together with the themes and categories that emerged from the analysis. The blue section represents autonomy and captures community nurses’ ability to organize their work independently, manage their time flexibly and make decisions aligned with their professional judgement. The orange section represents relatedness, highlighting the central role of interpersonal connections in community nursing, including collaboration with other healthcare professionals, meaningful interactions with clients and the development of trust and partnerships in caregiving. The green section represents competence, focussing on the professional skills and knowledge required to deliver high‐quality care. This dimension encompasses continuous professional development, problem‐solving and the ability to provide customized support to clients. Taken together, the figure provides a structured overview of the motivational factors that drive community nurses and that contribute to job satisfaction, professional growth and retention in the field.

### 5.1. Autonomy

#### 5.1.1. Shaping Everyday Practice on One’s Own Terms

Autonomy in the context of community nursing refers to nurses’ ability to act independently, make professional decisions and structure their work in alignment with their personal and professional values. For community nurses, autonomy means having the freedom to prioritize tasks, manage their schedules flexibly and tailor their care approaches to meet the unique needs of clients without external constraints. When autonomy is actively supported, community nurses experience increased engagement, greater responsibility and enhanced satisfaction in their professional roles, ultimately contributing to higher quality care delivery.

#### 5.1.2. Flexibility

##### 5.1.2.1. Organizing Time and Tasks Independently

Community nurses highly value the ability to independently organize their daily work, manage their time flexibly and make autonomous decisions regarding client care. This level of self‐determination not only enhances their sense of control over their work but also contributes to a better work–life balance.

A key aspect of this flexibility is the independent organization of everyday work, which allows community nurses to structure their tasks and responsibilities in a way that best suits their professional approach and the needs of their clients. Instead of following rigid schedules, they can prioritize tasks based on urgency and individual client needs. Additionally, flexible time management enables them to adjust their working hours according to personal and professional demands. This adaptability is particularly important in a role that requires responsiveness to clients’ varying needs while maintaining a sustainable work‐life balance.

“[…] but that way I can organise it myself, if I have to go home earlier on one day because I have to do something, I can stay longer another time, and the client visits, you organise them yourself, that actually works well, we’re in a situation where you have to be flexible with the project, we have appointments that you should attend, and yes, I actually like the open thing now, that I can organise it myself, that’s great […]” (CN4, Pos. 292‐298).

Moreover, community nurses appreciate the freedom to make autonomous decisions about how, when and in what manner they provide care. This flexibility fosters a more person‐centred approach, ensuring that interventions are tailored to the specific needs of each individual rather than being dictated by rigid institutional guidelines.

“[…] the client, the mother, was actually the centre of attention and the granddaughter came and the daughter‐in‐law and the son came and I have to say, yes, they were very, very pleased, as we said, because of the neurologist appointment, it’s often difficult with the care allowance application, that you have to fit it all in and so on and whether it would even work out, I said, I could also do a [standardized test] we have a new one now, yes, they were very pleased […]” (CN6, Pos. 38‐45).

#### 5.1.3. Professional Development

##### 5.1.3.1. Expanding Scope of Practice Through Self‐Directed Growth

The need for autonomy is also reflected in the way community nurses actively seek opportunities for personal and professional development, enabling them to take on new challenges and expand their expertise. Having the freedom to shape their own learning paths fosters motivation and a sense of ownership over their career progression. Professional development is categorized under autonomy rather than competence because, within the Austrian healthcare system, nurses’ scope of practice has traditionally been constrained by physician‐directed task delegation, often limiting nurses to operating below their formal qualifications [41–43]. Community nursing, by contrast, allows nurses to exercise their full professional skill set independently rather than primarily executing physician‐directed tasks, consistent with broader calls within Austrian primary care to expand nurses’ autonomous scope of practice [44]. It is this independent exercise and expansion of competence, rather than skill acquisition itself, that aligns professional development with the autonomy dimension in this study.

A key aspect of this is continuous learning and training, which allows community nurses to stay updated on the latest nursing knowledge, guidelines and best practices. Engaging in ongoing education helps them enhance their competencies and provide high‐quality care tailored to the evolving needs of their clients.

“You never stop learning, I really never stop learning” (CN2, Pos. 544)

Additionally, taking on new responsibilities is an important part of their professional growth. Many community nurses embrace the opportunity to step beyond routine care tasks, engaging in case management, health promotion initiatives and interprofessional collaboration. The ability to take on greater responsibilities fosters a sense of accomplishment and professional autonomy.

“I was able to deduce a bit from the job description and so on that it is compatible with home visits, but I have to say I underestimated the amount of knowledge we have actually acquired in just under a year […]” (CN7, Pos. 17‐20).

Moreover, community nurses value the opportunity for specialization and skill development, enabling them to refine their expertise in specific areas such as geriatric care, dementia support or preventive interventions. By deepening their knowledge and skills, they not only enhance their own career prospects but also improve the quality of care provided to their clients.

“[…] because I′ve been away from care for so long that I′m specialising in something, I′m now really very grateful to [my employer] for making it possible for me to do this with the dementia expert, because it also gives me security when I can really get involved in an area […]” (CN3, Pos. 323–327).

#### 5.1.4. Variety and Project Work

##### 5.1.4.1. Cocreating a New Field of Nursing

The opportunity to participate in a new field and work independently fosters a strong sense of autonomy and freedom of choice in the daily work of community nurses. Being involved in an evolving area of healthcare allows them to actively shape their roles while engaging in diverse and meaningful tasks.

A key element of this is the versatility of tasks, which ensures that community nursing remains dynamic and stimulating. Unlike traditional nursing roles that may be more rigid, community nursing allows nurses to engage in a wide range of activities, from direct client care to health promotion, administrative coordination and interprofessional collaboration. This variety enhances job satisfaction and prevents monotony.

“Very colourful, sometimes there are days when I am an office I sit in front of the PC, coordinate appointments, make phone calls, I think about how I can perhaps improve what I′m currently doing, then there are days when you get into the car in the morning and only get out in the evening, because in the morning there might be a network partner that you want to meet, then there are home visits, then there’s an office contact, so it′s really colourful […]” (CN14, Pos. 211‐220).

Another significant aspect is the opportunity for codesign within community nursing, which enables community nurses to contribute their insights and expertise to shaping the project’s structure, workflows and interventions. By having a say in decision‐making processes, they feel valued as professionals and are more motivated to drive innovation within the field.

“Yes, I just thought to myself, this is a project now, because I like to bring ideas into my work, and yes, that somehow motivated me for the job, that this is something I can be a part of, something that is built up from the beginning” (CN4, Pos. 48‐51).

Moreover, taking part in a new field of nursing in Austria presents new challenges that encourage growth and adaptability. Community nurses must navigate uncharted territory, develop creative solutions and refine their approaches based on real‐world experiences. This aspect not only strengthens their problem‐solving skills but also reinforces their autonomy by allowing them to take initiative and make informed decisions in their practice.

“[…] I knew it was a project, it was something new, it would also be an organisational part, so a lot to organise, coordinating, home visits, that′s roughly how I imagined it, and it′s also part of the public relations work, I couldn′t imagine much about that yet, but then I thought, yes, that′s something new, I′ll try it out, I′m open to it (CN3, Pos. 38‐43)

### 5.2. Competence

#### 5.2.1. Applying and Expanding Professional Skills Across a Broad Scope of Practice

Competence within community nursing refers to nurses’ ability to feel effective, skilled and confident in delivering high‐quality care. It encompasses their mastery in managing diverse and complex tasks, successfully addressing challenges and continuously enhancing their professional skills. For community nurses, direct client interactions, interprofessional collaboration and opportunities for continuous professional development significantly contribute to their sense of competence. Work environments that support ongoing learning, provide meaningful feedback and encourage skill expansion not only boost nurses’ motivation but also strengthen their capacity to positively impact patient outcomes and community health.

#### 5.2.2. Direct Contact With Clients

##### 5.2.2.1. Building Competence Through Hands‐On Client Care

Direct interaction with clients provides community nurses with a strong sense of competence, as it allows them to apply their expertise in a meaningful and impactful way. Through their daily work, they witness firsthand how their skills contribute to improving health outcomes and enhancing the well‐being of individuals in the community.

A crucial aspect of this is personal attention, which enables community nurses to address clients’ individual needs with care and empathy. Unlike in hospital settings, where time constraints often limit personalized interactions, community nurses can take the time to focus on each person’s unique situation, offering tailored advice and support.

“[…] that I can simply be there for people again, that I can take my time, because I worked at the hospital for a long time and there was no more time and that was always very difficult for me to work and that′s exactly what it is, the freedom to organise my time […]” (CN8, Pos. 9‐12).

Another fundamental element is listening and building trust, which strengthens the relationship between nurses and their clients. By providing a safe space for open conversations, community nurses help clients feel heard and understood. This trust is essential for effective health interventions, as it encourages individuals to openly share their concerns and adhere to recommended care plans.

“Through time, that′s actually for me, through time, that you really give them feeling during the home visits that we are there for you, regardless of whether it′s half an hour, two hours, and I realise that people really appreciate that, and that they can always call back, we are there, that they really have a contact person they trust […]” (CN6, Pos. 23‐27).

Furthermore, customized support is key to fostering a sense of competence in community nursing. By adapting their interventions to each client’s medical, social and emotional circumstances, nurses can provide individualized solutions that truly make a difference. This ability to tailor care to real‐life needs reinforces their professional confidence and satisfaction, further motivating them in their role.

“[…] I’m a nurse and of course I have a good overview of them [clients] and their lives and their homes and so on, I think that’s where people benefit the most, and that I also come more often […]” (CN5, Pos. 112‐114)

#### 5.2.3. Wide Range of Tasks

##### 5.2.3.1. Developing Well‐Rounded Expertise Across Professional Domains

The broad scope of responsibilities in community nursing strengthens nurses’ sense of competence, as they continuously apply and expand their skills across various professional domains. Their work extends beyond individual care, encompassing health promotion, administrative coordination and interprofessional collaboration, allowing them to develop a well‐rounded expertise.

A key aspect of this is the continuous alternation between administrative and practical tasks. Community nurses must seamlessly transition between hands‐on client care, documentation, case management and strategic planning. This balance enhances their professional competence by requiring both clinical expertise and organizational efficiency, ensuring that they are not limited to a single aspect of care but develop diverse competencies.

“What does my everyday life look like, my everyday life, yes, on the one hand I have home visits, on the other hand I have consultation hours, where people can simply come, ring the bell, and then you have to see what they need or perhaps they need to sit down together again and then part of it, a big part of it was networking, really a big part, where we introduced ourselves, who we are, what we do, that we get this out to the people a bit, then we will do crafts for Easter with elderly people today, for example, […], and then there′s the public relations part, with events […]” (CN3, Pos. 48‐60).

Additionally, the role presents a variety of challenges, requiring community nurses to adapt to new situations, solve problems and respond flexibly to changing client needs. Every day brings different experiences, from conducting home visits and supporting families to organizing educational events and navigating bureaucratic processes. Overcoming these challenges fosters resilience and reinforces their confidence in their professional abilities.

“[…] I’ve been in the hospital for a long time and the challenge is you don′t even know what all the applications for social security coverage and things like that are, so you have to do some research. […]” (CN8, Pos. 28‐29).

Another vital component is interprofessional co‐operation, which allows community nurses to work alongside physicians, social workers, allied health professionals and municipal service providers. Collaborating with experts from different fields broadens their knowledge base, strengthens their decision‐making skills and enhances the quality of care they can offer. Being an integral part of an interprofessional team validates their expertise and strengthens their professional identity.

“The Mayor anyway, citizen service, the cooperation is also very strong and very good, they also do a lot, because where do I go as a person who somehow needs help in that direction, either I go to the family doctor or I go to the local authority’s citizens’ service, that’s right, whether it’s about applications or whether it’s about care issues, I go to the family doctor or to the community to the local authority, that’s right, we are very strongly networked with them too […]” (CN9, Pos. 354‐359).

#### 5.2.4. Desire for a Sense of Purpose

##### 5.2.4.1. Gaining Confidence Through Meaningful Impact

Community nurses are highly motivated by the meaningfulness of their work, which reinforces their sense of competence. By seeing the direct impact of their efforts, they gain confidence in their abilities and feel a strong professional fulfilment. Their role extends beyond routine care, allowing them to actively shape the well‐being of individuals and the broader community.

A key aspect of this is improving the quality of life for their clients. Through health education, early interventions and individualized care, community nurses empower people to maintain their independence and enhance their overall well‐being. Witnessing these positive changes reinforces their belief that their work is valuable and that their expertise leads to tangible improvements. Additionally, their support for people in need is a crucial factor in their motivation. Many clients, particularly older adults and vulnerable populations, rely on community nurses as their primary source of healthcare guidance and emotional support. By assisting individuals who might otherwise struggle to access care, nurses experience a deep sense of professional purpose, knowing that their work directly contributes to better health outcomes and social inclusion.

“[…] and I was actually really looking forward to having more contact with people, and I had the feeling right from the start that I could do something really useful because I can steer people in a certain direction, give them advice and slowly lead them in the direction of prevention […]” (CN9, Pos. 23‐27)

Furthermore, community nurses recognize the long‐term impact on the community that their work creates. By engaging in preventive care, counselling and public health initiatives, they help shape a more resilient and health‐conscious society. Their role in fostering sustainable healthcare solutions and promoting well‐being at a systemic level reinforces their confidence and competence, knowing that their contributions extend beyond individual cases to influence broader social change.

“[…] a primary care centre in larger communities, where a community nurse is also on board, that would be my wish, and of course it should continue, logically, what we are doing makes a lot of sense […]” (CN5, Pos. 636‐639)

### 5.3. Relatedness

#### 5.3.1. Building Connection Through Collaboration and Community Ties

In the context of community nursing, relatedness refers to the nurses’ need to build meaningful social connections and experience a strong sense of belonging within their professional and community networks. It involves feeling valued, supported and deeply connected to clients, colleagues and local stakeholders. For community nurses, relatedness is fostered through close collaboration with interprofessional teams, personal connections to the community they serve and active contributions to local health initiatives. A robust sense of relatedness enhances their emotional well‐being, promotes professional engagement and motivates nurses to participate actively in improving community health outcomes.

#### 5.3.2. Community and Networking

##### 5.3.2.1. Strengthening Professional Relationships and Interprofessional Ties

Community nurses highly value collaboration and the ability to build strong professional relationships within the healthcare sector. Establishing connections with other professionals and services enhances their ability to provide comprehensive and effective care. The sense of social integration and support from colleagues play a crucial role in their motivation and job satisfaction.

A fundamental aspect of this is establishing a network of partners, which allows community nurses to work closely with healthcare providers, social workers and other relevant stakeholders. By developing strong partnerships, they can ensure that their clients receive holistic care, benefiting from a wide range of services tailored to their needs. These networks also serve as a valuable source of professional exchange and knowledge‐sharing.

“At the moment it’s still very much about networking, simply writing to groups, make contact and simply try to get into these networks somehow. […]” (CN2, Pos. 73‐75)

In addition, coordination between different stakeholders is essential for streamlining care processes and avoiding fragmentation. Community nurses act as key facilitators in linking medical professionals, social care providers and municipal services. Through effective coordination, they help create a seamless healthcare experience for clients, ensuring that interventions are well‐integrated and aligned with each individual’s needs.

“[…] and simply to get an overview of the care system in the region, because there is simply so much and somehow not even on the screen, if I may put it that way now, and is simply overwhelmed with the offer, and which one suits us now, exactly, so the case management, I think, is very big and useful for the relatives and for the clients […]” (CN13, Pos. 25).

Moreover, active participation in community projects strengthens their role as agents of change. By engaging in local health initiatives, prevention programs and outreach activities, they contribute to the overall well‐being of the community. These projects allow them to work beyond direct client care, influencing health policy and promoting sustainable healthcare models that address broader social determinants of health.

“[…] yes we work with the Healthy Community Project, we work together […]” (CN12, Pos. 158)

#### 5.3.3. Personal Connection to the Region

##### 5.3.3.1. Belonging to the Community Being Served

The sense of solidarity and belonging among community nurses is reinforced by the fact that many of them work within their own communities. Their strong connection to the people and the region fosters a deep commitment to their work and enhances the quality of care they provide.

A key aspect of this connection is their familiarity with the region and its people. Knowing the local culture, traditions and social structures allows community nurses to establish trust more easily with clients and their families. Their shared background with the community helps break down barriers and facilitates open communication, leading to a more personalized and effective approach to care.

“Exactly, I made house calls and started straight away, and because I knew the people personally, so there was no inhibition threshold, and it went well right from the first day, and it was euphoria straight away, yes so enthusiastic because it was well received […]” (CN1, pos. 57‐60).

Additionally, their commitment to the local community is reflected in their dedication to improving the well‐being of those they serve. Many community nurses feel a strong responsibility toward their hometowns and see their work as a way of giving back. This intrinsic motivation enhances job satisfaction and reinforces their sense of purpose.

“My motivation, above all, is actually the community, that I live in [name of community] and I′ve actually grown up here since I was a child, and I actually went to school in [name of community], I went to school in [name of community] for ten years and it′s actually almost my community, yes, and I know a lot of people and I′m actually also in the volunteer fire brigade and I just want to help people in my region.” (CN6, Pos. 5‐10).

Moreover, working within a familiar environment enables customization to local requirements. Community nurses can tailor their interventions based on the specific needs, challenges and resources available in their region. Whether adapting care plans to rural settings, addressing social disparities or collaborating with local organizations, they are uniquely positioned to implement context‐specific solutions that make a meaningful impact.

“Exactly, I’m from near [name of community], so now I’ve got to know [name of community], a little bit a bit and I already knew, for example, that [name of district], is more of a focal point where it’s difficult, and I knew that [name of district], was a posh neighbourhood, and I got to know the other areas through experience and simply discovered a little bit what’s actually going on there and what is needed” (CN10, pos. 279‐284).

#### 5.3.4. Contribution to Community

##### 5.3.4.1. Giving Back Through Social Engagement

Community nurses experience a strong sense of social involvement and motivation when they can actively contribute to society through their work. Their role extends beyond individual care, encompassing health education, prevention and support for families and caregivers. By raising awareness of nursing and healthcare within the community, they reinforce the importance of their profession and their role in shaping a healthier society.

Community nurses strive to enhance not just the physical well‐being of their clients but also their independence, dignity and overall life satisfaction. Through person‐centred care and continuous support, they help individuals maintain a higher level of autonomy and participation in daily life.

„[…] and also from our monthly cafés, which are not about drinking coffee and cake with senior citizens, but actually more about, firstly, to counteract loneliness, to create a kind of social space again, where is simply de facto zero and also do biography work […]” (CN10, Pos. 112‐116).

Additionally, health promotion and prevention play a crucial role in their work. By providing guidance on preventive healthcare measures, such as nutrition, physical activity and early detection of illnesses, they empower clients to take proactive steps toward maintaining their health. Their efforts help reduce the burden on acute healthcare services and contribute to a more sustainable healthcare system.

Another important dimension of their contribution is supporting informal carers. Many community nurses work closely with family members who take on caregiving responsibilities, offering them guidance, training and emotional support. By equipping informal carers with the knowledge and resources they need, nurses not only enhance the well‐being of the client but also prevent caregiver burnout and strengthen the overall caregiving network within the community.

“[…] but informal carers are actually always on their own, and they benefit a lot from that, and you also get positive feedback from them, and of course you take a lot off their shoulders, for example when a patient comes home, the informal carers are already overwhelmed because of the situations, and then they have to fill out a lot of forms or applications, and that′s where you help and support them, and that’s why I think they really benefit from us, because you take a bit of pressure off them, you give them a bit of freedom, that you listen to them and take some of their worries away” (CN15, pos. 81‐93)

**FIGURE 2:**
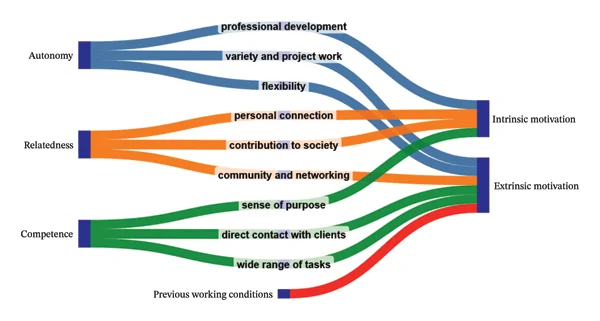
Mapping community nurses’ experiences to psychological needs and motivational drivers. Third analytical step: mapping to intrinsic and extrinsic forms of motivation.

SDT distinguishes between two main types of motivation: intrinsic motivation, which arises from personal interest or enjoyment, and extrinsic motivation, which is driven by external incentives such as rewards or sanctions. Figure 2 illustrates how various subcategories contribute to these forms of motivation.

Intrinsic motivation is particularly strengthened when the needs for autonomy, competence and relatedness are satisfied. In the diagram, subcategories such as desire for a sense of purpose, professional development, personal connection to the region and contribution to society flow into intrinsic motivation highlight their role in fostering long‐term engagement and job satisfaction.

Conversely, factors such as frustration with previous working conditions, variety and project work, community and networking, autonomy and flexibility, and direct contact with clients, shown here as leading to extrinsic motivation, demonstrate how external conditions influence professional behaviour. Notably, frustration with previous working conditions stands out as a key external driver as it was mentioned by the majority of community nurses. One participant described how long‐term exposure to systemic issues in healthcare led to exhaustion:

“[…] I was actually very motivated in my work or have been for a long time, you burn out or you just get a bit a little weary over time, because you simply see what is going wrong or what simply works badly […]” (CN5, pos. 9‐12).

Another community nurse highlighted the challenges of rigid scheduling and lack of work–life balance in their previous job

“[…] Jumping in, handing out, getting the duty rota late, no consideration for family problems family problems, that was very difficult […]” (CN8, pos. 16‐17).

While extrinsic motivators can play a role in initial engagement, SDT emphasizes that sustained motivation and satisfaction are best achieved when intrinsic needs are fulfilled.

## 6. Discussion

This study explored the experiences of community nurses in Austria’s pilot project through the lens of SDT. Findings highlight that autonomy is fostered through flexible scheduling, independent decision‐making and opportunities for professional development. Competence is reinforced by direct client interactions, interprofessional collaboration and skill expansion. Relatedness emerges from networking, regional familiarity and a sense of contribution to society. Additionally, intrinsic motivation, which is driven by a desire for meaningful work played a crucial role in job satisfaction, while extrinsic motivation, particularly frustration with previous work conditions, was a key driver for transitioning into community nursing.

These findings are consistent with international evidence on community nursing roles. In countries with well‐established community‐based care systems, community nurses similarly report high levels of professional autonomy, flexible working structures [45] and strong relational continuity with clients [46]. These features are considered central to both job satisfaction and retention [27, 47], suggesting that the Austrian pilot aligns with broader international developments in community‐oriented care.

The findings highlight autonomy as a central factor in sustaining motivation among community nurses, aligning with SDT, which identifies autonomy as a fundamental psychological need that fosters motivation, well‐being and job satisfaction [32, 33]. In the context of community nursing, the ability to independently organize work schedules and make client‐centred decisions contributed significantly to nurses’ intrinsic motivation. This is supported by existing international research showing that professional independence is associated with higher job satisfaction and lower burnout rates [48, 49]. Lautizi et al. [50] further emphasize that structural empowerment, including elements of autonomy, is a critical predictor of work engagement and retention in healthcare professions. Together, these findings confirm that workplace flexibility and decision‐making authority are essential conditions for fostering sustained motivation and commitment in community nursing.

Community nurses reported that direct client contact and interprofessional work enhanced their sense of competence. This is consistent with research showing that autonomous learning opportunities and diverse responsibilities improve professional confidence. Hoeve et al. [51] highlight that varied clinical experiences contribute to the development of professional identity and competence in nurses. The nurses’ ability to provide personalized care and build trusting relationships further strengthened their confidence in their professional expertise, similar to findings by Hartley et al. [52], which suggest that meaningful client interactions increase perceived competence in nursing professionals. Additionally, professional development opportunities, such as specialization in dementia care, provided a pathway for skill enhancement, further aligning with SDT principles that emphasize the importance of continuous learning for sustained motivation. This aligns with international literature highlighting the complexity and diversity of community nursing roles, which require both clinical expertise and coordination skills [53, 54]. International research indicates that opportunities to engage in holistic, person‐centred care and interprofessional collaboration contribute to nurses’ perceived competence and professional identity [55].

The results showed that strong professional networks and community ties significantly contributed to nurses’ motivation. Community nurses working in familiar regions reported a deep sense of belonging, reinforcing the idea that a strong connection to the community enhances job commitment. The interprofessional nature of their work also facilitated greater collaboration with physicians, social workers and municipal services, fostering an environment where they felt valued as part of a broader healthcare network [56]. The importance of relational continuity and community embeddedness is also reported in international research on community nursing [57, 58]. Strong connections with clients, families and local stakeholders are considered a key distinguishing feature of community‐based care [59] and are closely linked to both care quality and professional satisfaction [60].

Community nurses often face the misconception that they prefer managerial roles over direct patient care. However, evidence suggests that many nurses find significant satisfaction in direct interactions with patients and their caregivers [61, 62]. Furthermore, the implementation of primary nursing models, which emphasize continuity of care and the nurse–patient relationship, has been shown to enhance job satisfaction among nurses, indicating a preference for direct patient engagement over administrative duties [63].

The theme of frustration with previous working conditions emerged as a prominent overarching factor in our study, significantly influencing community nurses’ decisions to transition from traditional healthcare settings to community nursing. This theme stands apart from the psychological needs depicted in the sunburst visualization (Figure 1), as it does not represent a motivational need itself but rather a structural context in which motivation is shaped. Participants frequently described experiences of moral distress, excessive workloads, limited flexibility and burnout as central reasons for leaving their previous roles. These external pressures created a push factor that made the autonomy, flexibility and person‐centred focus of community nursing especially attractive. Due to its cross‐cutting nature and its relevance for both intrinsic and extrinsic motivation, frustration with previous working conditions is illustrated separately in Figure 2 to highlight its overarching role in shaping nurses’ career decisions. This finding aligns with existing literature indicating that adverse working conditions contribute substantially to nurse attrition. A comprehensive review identified poor working conditions, lack of career development opportunities, insufficient managerial support, work‐related stress and bullying as key factors driving nurses to leave the profession [22].

Moral distress impacts nurses’ intentions to leave their positions. Research indicates that 15% of nurses have reported leaving a job due to moral distress [64]. This distress often arises when nurses are unable to provide the level of care they deem necessary, leading to feelings of compromised integrity and professional dissatisfaction.

This underscores the urgent need for healthcare organizations to address the root causes of nurse dissatisfaction. Our findings show that nurses are not merely seeking new roles but are actively distancing themselves from systems that undermine their professional values. Strategies to reduce moral distress, manage workloads more effectively and establish supportive work environments are, therefore, essential, not only to retain staff but also to create conditions in which nurses can thrive. Addressing these issues is particularly relevant for fostering long‐term commitment to innovative care models such as community nursing.

### 6.1. Strengths and Limitations of the Work

Strengths: This study’s main strength lies in its systematic and transparent approach to data collection and analysis, including a structured interview guide, audio‐recording and verbatim transcription, and qualitative content analysis supported by MAXQDA to enhance consistency and traceability of coding (see Section 4.6). Recruiting community nurses from six different regions, and combining interviews with a one‐day shadowing period per participant, allowed for a rich, contextualized account of an emerging professional role, consistent with established criteria for trustworthiness in qualitative research [40]. Limitations: While this study provides valuable insights into the motivations of community nurses in Austria, some limitations must be acknowledged. Not all interviews were conducted face to face; four of the 15 interviews were conducted online via Zoom because of geographical distance, illness‐related absences or scheduling constraints. Video‐mediated interviews may have limited the researchers’ ability to observe nonverbal cues and could have affected rapport compared with in‐person interviews [65]; no systematic differences in the depth or content of responses were, however, observed between the two interview formats during analysis. As participants were recruited from within an ongoing pilot project whose continuation depended on demonstrating its value, some responses may have been shaped by a wish to portray the project favourably, and the possibility of social desirability bias cannot be ruled out. The study only included community nurses from a single provider within the Austrian community nursing pilot project. As the pilot project allows different providers to implement varying approaches [11], the findings may not be fully representative of all community nursing models within Austria. The selected provider followed the international framework “Public Health Intervention Wheel” [66] for community nursing, emphasizing an open, counselling‐focused and empowering approach with minimal hands‐on care. This specific implementation could influence the perspectives of participating nurses and may not reflect the experiences of those working under different frameworks. Looking more broadly at the overall pilot project, its short duration and its discontinuation mean that long‐term impacts on both nurses and their clients remain uncertain. The findings reflect initial experiences and motivations but may not capture challenges that could emerge as the project develops further. Future research should explore the sustainability of community nursing models and assess perspectives from multiple providers to gain a more comprehensive understanding of the role and impact of community nurses in Austria.

### 6.2. Recommendations for Further Research

Future studies should investigate the long‐term effects of autonomy, competence and relatedness on job retention in community nursing. Longitudinal studies are needed to assess how sustained support for these psychological needs influences retention rates among community nurses. Understanding these dynamics can inform strategies to maintain a stable nursing workforce. Comparative research should be conducted across different healthcare systems to assess how varying structural and policy frameworks impact nurse motivation and retention. Such studies can identify best practices and inform policy decisions to enhance nurse satisfaction globally. Additionally, future research should explore the perspectives of clients and other stakeholders to understand how community nursing influences healthcare outcomes. Investigating how community nursing impacts healthcare outcomes from the viewpoints of clients and other stakeholders can provide a comprehensive understanding of its effectiveness. This approach ensures that nursing practices align with patient needs and expectations, ultimately improving care quality. By addressing these areas, healthcare policymakers and institutions can develop targeted strategies to enhance nurse satisfaction, improve retention and deliver high‐quality patient care.

### 6.3. Implications for Policy and Practice

The conclusion of EU funding in 2024 marks a critical turning point for community nursing in Austria. Despite positive evaluation results from both our qualitative study and the Austrian Public Health Institute’s final report [11], there has been no national rollout. Instead, many regions have discontinued the service altogether. This represents a missed opportunity to establish a sustainable, preventive health infrastructure at a time when Austria’s ageing population and growing care needs call for exactly such innovative, relationship‐based models. Without structural integration, the substantial investments in nurse training, community engagement and implementation risk being lost, eroding the trust and continuity that were central to the intervention’s success.

To safeguard these gains and build on the strong evidence base, Austria needs a coordinated national strategy for community nursing. Such a strategy should be anchored in legislation, supported by long‐term budgetary commitments and aligned with the World Health Organization’s priorities for strengthening primary and preventive care [16]. Embedding community nursing as a permanent element of the health system would not only preserve the professional expertise and motivation cultivated during the pilot phase but also ensure equitable access to preventive services across regions. Policymakers now have the opportunity and responsibility to act decisively, transforming a time‐limited project into a lasting pillar of Austria’s public health landscape.

## 7. Conclusion

This study highlights that autonomy, competence and relatedness are central to sustaining motivation and job satisfaction among community nurses in Austria’s pilot project. By integrating SDT principles into workforce policies, healthcare systems can foster environments that enhance engagement, reduce turnover and ultimately improve patient care. Moreover, the findings demonstrate that community nursing is an attractive and fulfilling career path for nurses in Austria, offering a work environment that prioritizes flexibility, professional development and meaningful interpersonal connections. The pilot project has shown that when nurses experience autonomy in their decision‐making, competence in their professional roles and strong relatedness with clients and colleagues, they are more likely to remain engaged and satisfied in their work. Given the increasing need for community‐based healthcare services, policymakers should consider expanding and sustaining community nursing roles as a key strategy to address workforce shortages and improve primary healthcare delivery. The study contributes to ongoing discussions on optimizing community nursing as an essential component of Austria’s healthcare system, ensuring both nurse retention and improved health outcomes for the population.

## Funding

This study did not receive a dedicated external research grant.

## Disclosure

Data collection was carried out by the first author (CF) within the scope of her professional employment at the University of Applied Sciences Campus Vienna; data analysis and manuscript preparation were conducted independently as part of her doctoral studies.

## Conflicts of Interest

The authors declare no conflicts of interest.

## Supporting Information

Additional supporting information can be found online in the Supporting Information section.

## Supporting information


**Supporting Information** Supporting Information for this article includes a completed Standards for Reporting Qualitative Research (SRQR) checklist.

## Data Availability

The data supporting the findings of this study are not publicly available due to their containing information that could compromise the privacy and anonymity of research participants. Anonymized excerpts may be made available from the corresponding author upon reasonable request, subject to the approval of the responsible ethics committee.
