# Supplementary material for: Motivations to Enter and Remain in Community Nursing: Implications for Workforce Sustainability
Source: J Nurs Manag. 2026 Sep 22;2026:7221720. doi: 10.1155/jonm/7221720 (PMC13598440; doi:10.1155/jonm/7221720)
Supplement: Supplementary file 1 [file JONM-2026-7221720-s001.docx]

**Standards for Reporting Qualitative Research (SRQR) Checklist**

| **SRQR Item** | **Description** | **Location in Manuscript** |
| --- | --- | --- |
| **S1 Title** | Concise description of the nature and topic of the study, identifying the qualitative approach | Title page, p. 1 |
| **S2 Abstract** | Summary of background, purpose, methods, results, and conclusions | Abstract, pp. 1–2 |
| **S3 Problem formulation** | Description and significance of the problem and review of relevant literature | Introduction, pp. 2–5 |
| **S4 Purpose / research question** | Purpose of the study and specific objectives | Section 3.1 Aim(s), p. 5 |
| **S5 Qualitative approach and paradigm** | Qualitative approach and guiding theory | Section 2 Background (theoretical framework), pp. 4–5; Section 4.1 Design, p. 5 |
| **S6 Researcher characteristics and reflexivity** | Researchers’ characteristics and reflexivity | Section 4.6 Rigor and Reflexivity, p. 8 |
| **S7 Context** | Setting and contextual factors | Section 4.2 Study Setting and Recruitment, p. 6 |
| **S8 Sampling strategy** | Sampling method, inclusion criteria, and rationale | Section 4.2 Study Setting and Recruitment, p. 6 |
| **S9 Ethical issues** | Ethics approval and informed consent | Section 4.5 Ethical Considerations, p. 7 |
| **S10 Data collection methods** | Types of data and data collection procedures | Section 4.3 Data Collection, p. 6 |
| **S11 Data collection instruments and technologies** | Description of interview guide and tools | Section 4.3 Data Collection, p. 6 |
| **S12 Units of study** | Number and characteristics of participants | Section 5.1 Characteristics of Participants, p. 8 |
| **S13 Data processing** | Transcription, data management, and anonymization | Sections 4.3 Data Collection; 4.5 Ethical Considerations, pp. 6–7 |
| **S14 Data analysis** | Analytic process and framework | Section 4.4 Data Analysis, pp. 6–7 |
| **S15 Techniques to enhance trustworthiness** | Strategies to enhance credibility and rigor | Section 4.6 Rigor and Reflexivity, p. 8 |
| **S16 Synthesis and interpretation** | Main findings and interpretations | Section 5 Findings, pp. 8–17 |
| **S17 Links to empirical data** | Use of quotations and evidence | Section 5 Findings, pp. 8–17 |
| **S18 Integration with prior work and implications** | Integration with literature and contribution | Section 6 Discussion, pp. 18–20 |
| **S19 Limitations** | Trustworthiness and limitations | Section 6.1 Strengths and Limitations, p. 20 |
| **S20 Conflicts of interest** | Declaration of conflicts | Section 8.2 Conflict of Interest, p. 21 |
| **S21 Funding** | Sources of funding and role of funders | Section 8.3 Funding, p. 21 |
